# Supplementary figures and images for: CircRNA_012164/MicroRNA-9-5p axis mediates cardiac fibrosis in diabetic cardiomyopathy
Source: PLoS One. 2024 Jul 23;19(7):e0302772. doi: 10.1371/journal.pone.0302772 (PMC11265710; doi:10.1371/journal.pone.0302772)

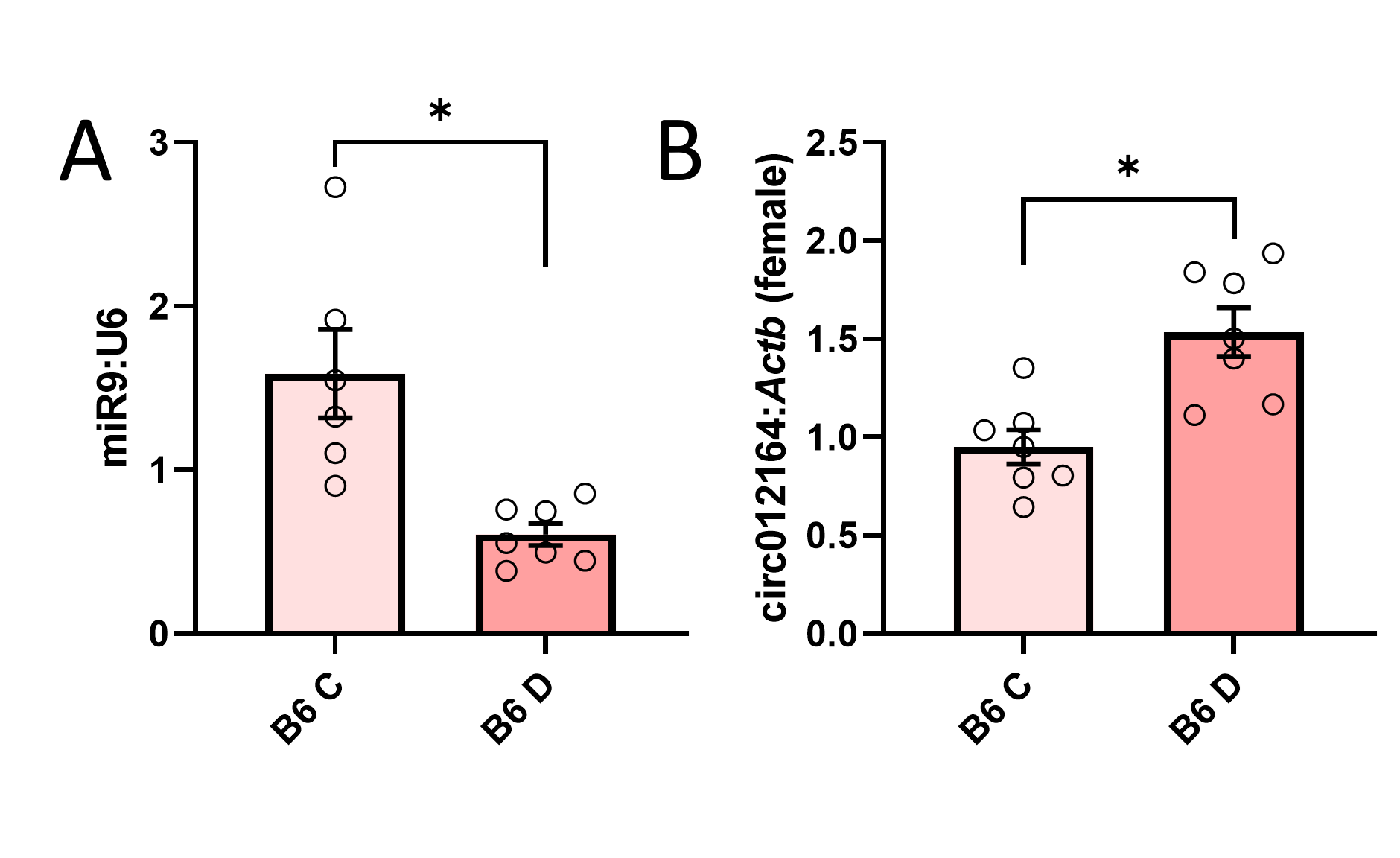

Supplement: S1 Fig — (TIF) [file pone.0302772.s001.tif]

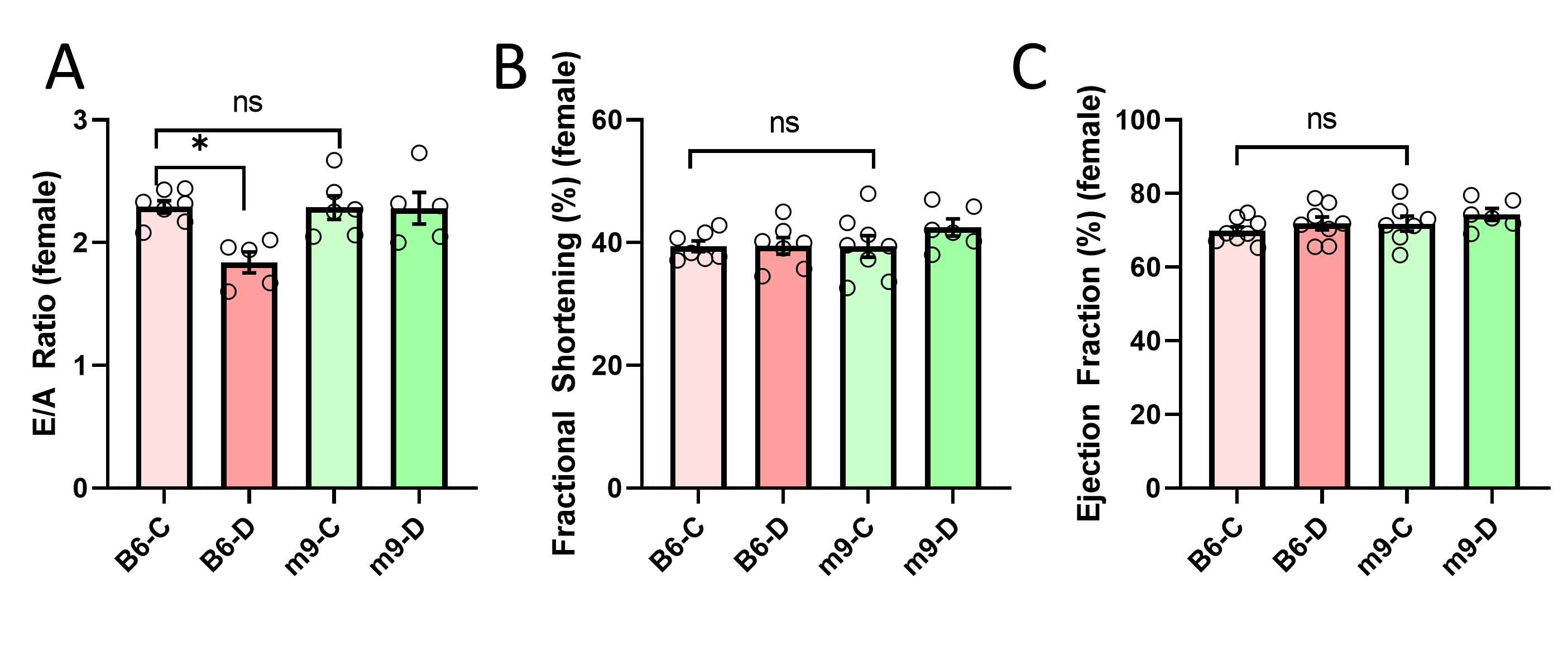

Supplement: S2 Fig — A) E/A ration, B) fractional shortening percent and C) ejection fraction percent in female mouse echocardiography. No differences were shown between male and female mice. (TIF) [file pone.0302772.s002.tif]

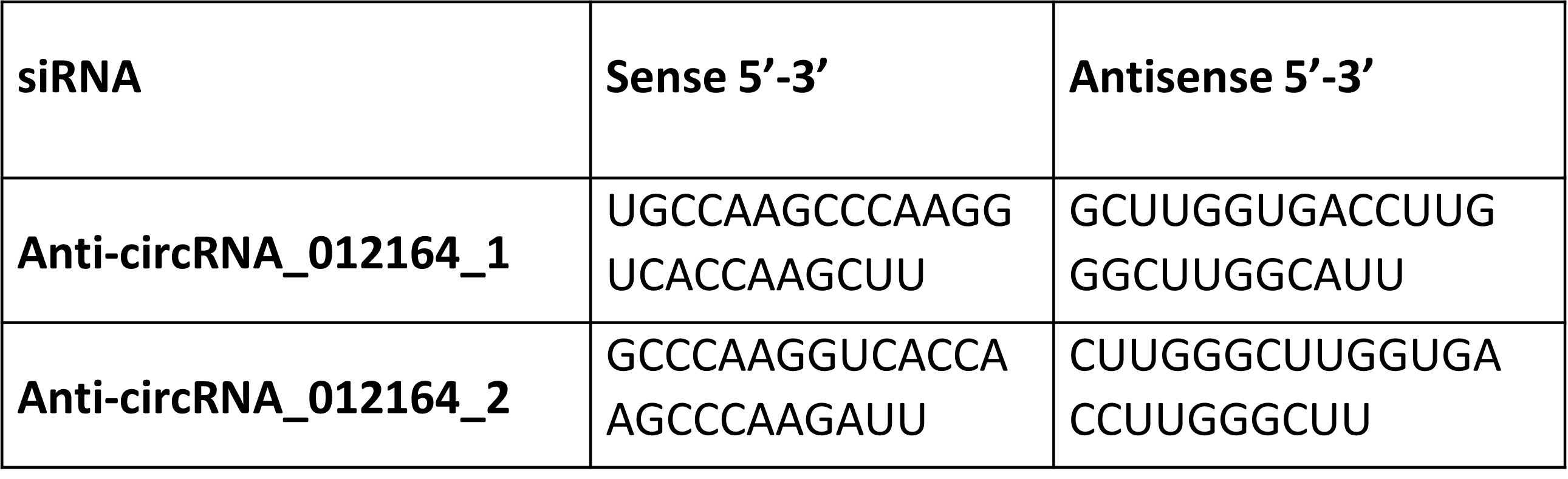

Supplement: S1 Table — (TIF) [file pone.0302772.s003.tif]
